# *Acinetobacter baumannii* nosocomial pneumonia: is the outcome more favorable in non-ventilated than ventilated patients?

**DOI:** 10.1186/1471-2334-13-142

**Published:** 2013-03-19

**Authors:** Ya-Sung Yang, Yi-Tzu Lee, Tsai-Wang Huang, Jun-Ren Sun, Shu-Chen Kuo, Chin-Hsuan Yang, Te-Li Chen, Jung-Chung Lin, Chang-Phone Fung, Feng-Yee Chang

**Affiliations:** 1 Division of Infectious Diseases and Tropical Medicine, Department of Internal Medicine, Tri-Service General Hospital, National Defense Medical Center, Taipei, Taiwan; 2 Institute of Clinical Medicine, School of Medicine, National Yang-Ming University, Taipei, Taiwan; 3 Emergency Department, Taipei Veterans General Hospital, Taipei, Taiwan; 4 Division of Thoracic Surgery, Department of Surgery, Tri-Service General Hospital, National Defense Medical Center, Taipei, Taiwan; 5 Clinical Microbiology Laboratory Division of Clinical Pathology, Tri-Service General Hospital, National Defense Medical Center, Taipei, Taiwan; 6 National Institute of Infectious Diseases and Vaccinology, National Health Research Institutes, Miaoli County, Taiwan; 7 Department of Health, Centers for Disease Control, Taipei, Taiwan

**Keywords:** Acinectobacter baumannii, Pneumonia, Hospital acquired, Ventilator

## Abstract

**Background:**

*Acinetobacter baumannii* hospital-acquired pneumonia (HAP) is associated with a high mortality worldwide. Non-ventilated patients with HAP (NVHAP) caused by nosocomial pathogens are reported to have a more favorable outcome than those with ventilator-associated pneumonia (VAP). The current study was designed to determine whether bacteremic patients with *A. baumannii* NVHAP also have a lower mortality than those receiving assisted ventilation.

**Methods:**

This retrospective 10-year study was conducted at a 2900-bed teaching hospital located in Northern Taiwan. The population consisted of 144 patients with *A. baumannii* bacteremia and HAP. Of these 96 had VAP and 48 had NVHAP. Charts were reviewed for demographic characteristics, comorbidities, clinical manifestations, antimicrobial susceptibility, and 14-day mortality. Clonal relationships were determined by molecular typing.

**Results:**

There were no significant differences between the two groups in comorbidities (Charlson scores). Patients with NVHAP were more likely to have developed bacteremia earlier, outside the ICU and undergone fewer invasive procedures. They had significantly lower APACHE II scores, fewer bilateral pneumonias and lower rates of antimicrobial resistance. No specific clones were identified in either group. The unadjusted (crude) 14-day mortality rates were not significantly different between the groups (NVHAP 43.8% vs. VAP 31.3%, *p* = 0.196). The adjusted 14-day mortality risk was significantly lower in ventilator-assisted patients (odds ratio = 0.201; 95% confidence interval = 0.075-0.538; *p* = 0.001).

**Conclusions:**

Patients with bacteremic NVHAP and VAP caused by *A. baumannii* had similar crude mortality rates, but on logistic regression analysis those receiving ventilator assistance had a significantly lower mortality. This may have been due to better airway protection, more intensive monitoring with earlier diagnosis and treatment in patients with VAP, greater innate susceptibility to infection in those with NVHAP and differences in the virulence of *A. baumannii*.

## Background

The three most clinically relevant, but phenotypically undifferentiated *Acinetobacter* species, *Acinetobacter baumannii*, *Acinetobacter nosocomialis* (formerly *Acinetobacter* genomic species 13TU), and *Acinetobacter pittii* (formerly *Acinetobacter* genomic species 3), have emerged as important nosocomial pathogens [1]. The prevalence of health care associated infections caused by *Acinetobacter* is increasing among patients in intensive care units (ICUs) and immunocompromised hosts [2-5]. The most common clinical condition associated with these microorganisms is hospital-acquired pneumonia (HAP), particularly for patients receiving mechanical ventilator assistance [6].

Among the three *Acinetobacter* species, *A. baumannii* is associated with a poorer outcome and higher rates of antimicrobial resistance [7]. Several *A. baumannii* virulence factors have been identified. These include CsuA/BABCDE, a chaperone-usher pili assembly system, the siderophore-mediated iron acquisition system and outer membrane protein A (OmpA) [8,9]. The mortality rates for bacteremia caused by *A. baumannii* range from 29.8 to 58.6% [7,10,11]. It has been previously reported that non-ventilated patients with HAP (NVHAP) caused by nosocomial pathogens have a better outcome than those with ventilator-associated pneumonia (VAP) [12-14]. It is not known whether this applies to *A. baumannii* as well. The current retrospective study was designed to determine whether patients with *A. baumannii* bacteremic NVHAP have a better outcome than those with bacteremic VAP.

## Methods

### Study population

This study was conducted at Taipei Veterans General Hospital (T-VGH) during a ten-year period from January 2000 to December 2009. T-VGH is a 2900-bed tertiary-care teaching hospital located in Taipei, Taiwan. The data were analyzed at the Tri-Service General Hospital (TSGH), National Defense Medical Center in Taipei, Taiwan.

Charts were reviewed for all patients with at least one positive blood culture for *A. baumannii* who had symptoms and signs of infection. The inclusion criteria for *A. baumannii* pneumonia [15] consisted of: a) at least one positive respiratory sample (sputum, endotracheal aspirate, or broncho-alveolar lavage [BAL]) for *A. baumannii* obtained within 48 hours before or after the first positive blood culture; b) a clinical course compatible with pneumonia, including symptoms of acute respiratory infection and acute infiltrates on a chest radiograph; and c) the positive blood culture could not be attributed to another source of infection. NVHAP was defined as pneumonia that occurred 48 hours or more after admission in non-ventilated patients. VAP was defined according to the 2005 American Thoracic Society/Infectious Diseases Society of America (ATS/IDSA) guidelines [16] as pneumonia that occurred more than 48 hours after endotracheal intubation. The quantitative microbiologic criteria for the diagnosis of HAP required a bacterial count exceeding a threshold of 10^3^ cfu/mL in a protected specimen brushing, 10^4^ or 10^5^ cfu/mL in a BAL fluid specimen or 10^6^ cfu/mL in an endotracheal aspirate [16]. Patients <18 years of age and those with incomplete medical records were excluded. All the clinical samples were taken as part of standard care. The standards of patient care did not significantly change during the study period. Mechanical ventilated patients not admitted to an ICU were treated in a respiratory care center, respiratory ward or common ward. The protocol was approved by the T-VGH and TSGH Institutional Review Board (approval number: 2011-10-012IC and 2-101-05-074) with a waiver for informed consent.

### Data Collection

Medical records were reviewed to extract clinical information, including demographic characteristics, underlying diseases, Charlson comorbidity score [17], duration of stay in an ICU, hospital stay, administration of individual antimicrobials, the presence of a ventilator, central venous catheters, a nasogastric tube, or a foley catheter at the time of onset of bacteremia.

Chronic lung diseases other than chronic obstructive pulmonary disease (non-COPD chronic lung disease) included asthma, bronchiectasis, pulmonary fibrosis, and chronic pulmonary tuberculosis. Immunosuppressive therapy was defined as receipt of cytotoxic agents within 6 weeks, corticosteroids at a dosage equivalent to or higher than 10 mg of prednisolone daily for more than 5 days within 4 weeks, or other immunosuppressive agents within 2 weeks prior to the onset of bacteremia. Neutropenia was defined as an absolute neutrophil count <0.5×10^9^ neutrophils/L. Recent surgery was defined as operations performed within 4 weeks prior to the onset of bacteremia. Chronic kidney disease was defined as an estimated glomerular filtration rate <60 mL/min/1.73 m^2^ for at least 3 months prior to admission. The severity of illness was evaluated using the acute physiology and chronic health evaluation II (APACHE II) score [18] within 24 hours prior to bacteremia onset.

Antimicrobial therapy was mainly based on the ATS/IDSA guidelines (combined with the clinical judgment of individual primary care physicians) [16]. Appropriate antimicrobial therapy was defined as administration of at least one antimicrobial agent, to which the causative pathogen was susceptible, within 48 hours after the onset of bacteremia, with an approved route and dosage for end organ function. Antimicrobial therapy that did not meet this definition was considered as inappropriate. Monotherapy with an aminoglycoside was not considered to be appropriate therapy. The primary outcome measure was all-cause 14-day mortality following the onset of bacteremia.

### Microbiological Studies

Only the first blood culture from patients with two or more positive blood cultures was included in the analysis. The presumptive identification of the isolates to the level of *A. baumannii* was performed with the API ID 32 GN system (bioMérieux, Marcy l’Etoile, France) or Vitek 2 system (bioMérieux, Marcy l’Etoile, France). A multiplex-PCR method was used to identify *A. baumannii* to the genomic species level [19]. Antimicrobial susceptibilities were determined by the agar dilution method according to the Clinical Laboratory Standards Institute (CLSI) [20]. Multidrug resistance was defined as resistance to three or more of the following classes of antimicrobial agents: anti-pseudomonal cephalosporins, anti-pseudomonal carbapenems, ampicillin/sulbactam, fluoroquinolones, and aminoglycosides [21].

### Molecular typing

The clonal relationships of the clinical isolates were determined by pulsed-field gel electrophoresis (PFGE). PFGE of ApaI-digested genomic DNA was performed using the Bio-Rad CHEF-Mapper apparatus (Bio-Rad Laboratories, Hercules, CA, USA). DNA restriction patterns were interpreted according to the criteria of Tenover et al. [22] and cluster analysis was performed using BioNumerics version 5.0 (Applied Maths, Sint-Martens-Latem, Belgium) and the unweighted pair-group method with arithmetic averages (UPGMA). The Dice correlation coefficient was used with a tolerance of 1% in order to analyze any similarities between banding patterns. In brief, isolates showing more than three DNA fragment differences and a similarity of <80% following dendrogram analysis were considered to represent different pulsotypes.

### Statistical analysis

To assess differences, the chi-square test with Yate’s correction or Fisher’s exact test was used to compare the discrete variables; the Student’s t-test or Mann–Whitney rank sum test was used to analyze continuous variables. Logistic regression models were used to explore independent risk factors for 14-day mortality. Univariate analyses were performed separately for each of the risk factor variables to ascertain the odds ratio (OR) and 95% confidence interval (CI). All biologically plausible variables with a *p* value of ≤ 0.20 in the univariate analysis exhibited by at least 10% of the patients were considered for inclusion in the logistic regression model for the multivariate analysis. A backward selection process was utilized. Time to mortality was analyzed using Kaplan-Meier survival analysis. A *p* value <0.05 was considered statistically significant. All the analyses were processed with Statistical Package for the Social Sciences (SPSS) software version 18.0 (SPSS, Chicago, IL, USA).

## Results

During the study period 357 patients were found to have had at least one episode of *A. baumannii* bacteremia. We excluded 209 patients with polymicrobial bacteremia (89 patients) and those with positive blood culture attributable to another source of infection (120 patients). The final population that met the criteria for entry into the study consisted of 148 patients of *A. baumannii* bacteremic pneumonia. Four patients were further excluded because of incomplete medical records. Among 144 patients, 48 had NVHAP and 96 had VAP. In addition to the clinical features and radiographic findings that were compatible with diagnosis of pneumonia, 93 VAP patients had positive cultures from endotracheal aspirates, three from BAL, and all NVHAP patients from sputum specimens.

The demographic and clinical characteristics of the study patients are summarized in Table 1. Compared to VAP patients, NVHAP patients acquired their infections less frequently in an ICU, earlier after admission, received intensive care for pneumonia less frequently and received fewer procedures. Nineteen NVHAP patients (12 acquired pneumonia in an ICU and 7 outside an ICU) who received intensive care in an ICU had mechanical ventilation. Comorbidities and laboratory parameters were similar between 2 groups, except that NVHAP patients had received a fewer recent surgical procedures.

**Table 1:** **Demographic and clinical characteristics of patients with bacteremic hospital-acquired pneumonia caused by *Acinetobacter baumannii***^**a**^

**Demographic or characteristic**	**Non-ventilator-associated (n = 48)**	**Ventilator-associated (n = 96)**	***p *Value**
	**n (% or IQR**^**a**^**)**		
Age in year	74 (54.8-81.8)	74 (59.0-80.0)	0.944
Gender, male	40 (83.3)	73 (76.0)	0.43
Acquired in ICU	12 (25.0)	84 (87.5)^b^	<0.001
ICU admission after pneumonia	19 (39.6)^c^	86 (89.6)	<0.001
Days of hospitalization prior to bacteremia	14.5 (3.2-3.6)	22.5 (12.0-40.8)	0.021
Comorbidity			
Charlson comorbidity score	3 (2.0-5.0)	3 (2.0-5.0)	0.861
Hypertension	22 (45.8)	47 (49.0)	0.86
Coronary artery disease	14 (29.2)	15 (15.6)	0.091
Congestive heart failure	6 (12.5)	19 (19.8)	0.392
Cerebral vascular disease	7 (14.6)	27 (28.1)	0.111
COPD	12 (25.0)	27 (28.1)	0.842
Non-COPD chronic lung disease	5 (10.4)	15 (15.6)	0.551
Alcohol	3 (6.3)	13 (13.5)	0.302
Liver cirrhosis	4 (8.3)	5 (5.2)	0.481
Chronic kidney disease	15 (31.3)	32 (33.3)	0.95
Type 2 diabetes	10 (20.8)	35 (36.5)	0.086
Collagen vascular disease	4 (8.3)	7 (7.3)	1
Usage of immunosuppressants	18 (37.5)	32 (33.3)	0.757
Usage of corticosteroids	7(14.6)	23 (24.0)	0.276
Neutropenia	4 (8.3)	7 (7.3)	1
Malignancy	22 (45.8)	28 (29.2)	0.073
Hematologic malignancy	7 (14.6)	6 (6.3)	0.125
Solid malignancy	17 (35.4)	23 (24.0)	0.211
Previous shock	8 (16.7)	27 (28.1)	0.192
Recent surgery	8 (16.7)	34 (35.4)	0.032
Trauma	0 (0)	4 (4.2)	0.301
Procedures^d^			
Abdominal drain	1 (2.1)	5 (5.2)	0.658
Arterial line	5 (10.4)	36 (37.5)	0.001
Central venous catheter	22 (45.8)	71 (74.0)	0.002
Pulmonary artery catheter	6 (12.5)	20 (20.8)	0.319
Foley catheter	28 (58.3)	78 (81.3)	0.006
Hemodialysis	4 (8.3)	10 (10.4)	0.774
Nasogastric tube	35 (72.9)	93 (96.9)	<0.001
Thoracic drain	0 (0)	7 (7.3)	0.095
Total parental nutrition	1 (2.1)	8 (8.3)	0.273
Tracheostomy	7 (14.6)	16 (16.7)	0.936
Shock^d^	29 (60.4)	58 (60.4)	1
APACHE II score^d^	24 (17.3-31.8)	27 (21.3-34.8)	0.015
Radiological features			
Bilateral radiologic involvement	27 (56.3)	81 (84.4)	0.001
Pleural effusion	20 (41.7)	32 (33.3)	0.425
Appropriate antimicrobial therapy	20 (41.7)	28 (29.2)	0.189
Combination antimicrobial therapy	4 (8.3)	7 (7.3)	1
Outcome			
14-day mortality	21 (43.8)	30 (31.3)	0.196

^a^Data are median value (interquartile range) for continuous variables and number of cases (%) for categorical variables. IQR = interquartile range; ICU = intensive care unit; COPD = chronic obstructive pulmonary disease; APACHE = Acute Physiologic and Chronic Health Evaluation.

^b^Other patients were cared in a respiratory care center, respiratory ward, or common ward.

^c^Twelve acquired pneumonia in an ICU and 7 outside an ICU, all of them received mechanical ventilation after pneumonia.

^d^At the time the blood culture was obtained.

The Charlson comorbidity scores on hospital admission were similar for the two groups. Patients with NVHAP had significantly lower APACHE II scores at bacteremia onset and less often presented with bilateral lung involvement on radiological examination at the time of onset of bacteremia.

**Figure 1:**
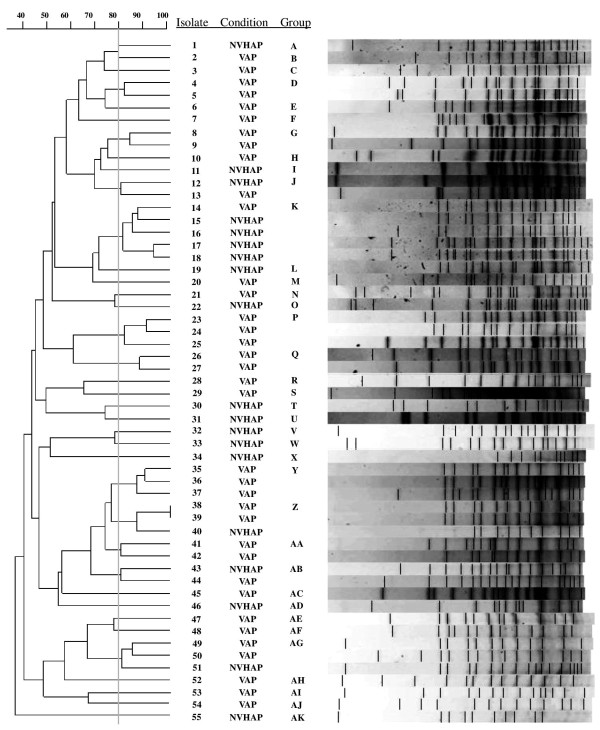
**Pulsed-field gel electrophoresis of 55 representative strains of *Acinetobacter baumannii *from patients with bacteremic nosocomial pneumonia.** NVHAP = non-ventilated hospital-acquired pneumonia; VAP = ventilator-associated pneumonia.

To determine the molecular epidemiology of the causative pathogens, 55 (38.2%) of the *A. baumannii* isolates were randomly selected for PFGE analysis. PGFE revealed 37 different pulsotypes (A to AK, Figure 1). Among the patients with NVHAP, there were 19 isolates in 16 pulsotypes: A (n = 1), I (n = 1), J (n = 1), K (n = 4), L (n = 1), O (n = 1), T (n = 1), U (n = 1), V (n = 1), W (n = 1), X (n = 1), Z (n = 1), AB (n = 1), AD (n = 1), AG (n = 1), and AK (n = 1). In VAP patients, there were 36 isolates in 26 pulsotypes: B (n = 1), C (n = 1), D (n = 2), E (n = 1), F (n = 1), G (n = 2), H (n = 1), J (n = 1), K (n = 1), M (n = 1), N (n = 1), P (n = 3), Q (n = 2), R (n = 1), S (n = 1), Y (n = 3), Z (n = 2), AA (n = 2), AB (N = 1), AC (n = 1), AE (n = 1), AF (n = 1), AG (n = 2), AH (n = 1), AI (n = 1), and AJ (n = 1).

The antimicrobial susceptibility profiles of the clinical isolates of *A. baumannii* are shown in Table 2. Isolates from NVHAP patients exhibited significantly lower rates of resistance to all antimicrobials tested. The initial antimicrobial agents used in both group are summarized in Table 3. The selection of antimicrobial agents was similar between both groups. Anti-pseudomonal carbapenems were the most commonly used agents in both groups, followed by anti-pseudomonal cephalosporins and penicillins. More NVHAP than VAP patients received an appropriate antimicrobial therapy, but the differences were not significant (41.7% vs. 29.2%, *p* = 0.189).

**Table 2:** Comparison of antimicrobial susceptibilities of blood isolates in patients with non-ventilator and ventilator-associated hospital-acquired pneumonia

	**Resistance, n (%)**		
**Antimicrobial agent**	**Non-ventilator associated (n = 48)**	**Ventilator-associated (n = 96)**	***p *Value**
Amikacin	27 (56.3)	86 (89.6)	<0.001
Gentamicin	32 (68.1)	88 (93.6)	<0.001
Ceftazidime	30 (63.8)	93 (96.9)	<0.001
Cefepime	22 (45.8)	73 (76.0)	0.001
Piperacillin/tazobactam	25 (52.1)	77 (80.2)	0.001
Ampicillin/sulbactam	17 (36.2)	62 (65.3)	0.002
Ciprofloxacin	33 (68.8)	93 (96.9)	<0.001
Imipenem	13 (27.1)	47 (49.0)	0.020
Multidrug resistance^a^	31 (64.6)	94 (97.9)	<0.001

^a^Definition: resistance to three or more of the following classes of antimicrobial agents: anti-pseudomonal cephalosporins, anti-pseudomonal carbapenems, ampicillin/sulbactam, fluoroquinolones, and aminoglycosides.

**Table 3:** Comparison of initial antimicrobial agents use in patients with non-ventilator and ventilator-associated hospital-acquired pneumonia

**Main agents used**	**Group, n (%)**		***p *Value**
	**Non-ventilator associated (n = 48)**	**Ventilator-associated (n = 96)**	
Anti-pseudomonal penicillins^a^	5 (10.4)	10 (10.4)	1.000
Anti-pseudomonal cephalosporins^b^	12 (25.0)	18 (18.8)	0.514
Anti-pseudomonal fluoroquinolones^c^	4 (8.3)	4 (4.2)	0.441
Anti-pseudomonal carbapenems^d^	14 (29.2)	29 (30.2)	1.000
Ampicillin/sulbactam or sulbactam	5 (10.4)	13 (13.5)	0.789
Non-anti-pseudomonal β-lactamase^e^	6 (12.5)	16 (16.7)	0.682
Miscellaneous^f^	2 (4.2)	6 (6.3)	0.719

^a^Including piperacillin, piperacillin/tazobactam, and ticarcillin/clavulanate.

^b^Including cefoperazone, ceftazidime, cefepime, and cefpirome.

^c^Including ciprofloxacin and levofloxacin.

^d^Including imipenem and meropenem.

^e^Including penicillin, amoxicillin/clavulanate, cefazolin, cefuroxime, cefotaxime, cefmetazole, and flomoxef.

^f^Including teicoplanin or clindamycin plus amikacin.

The 14-day mortality rate was slightly higher in NVHAP patients than those with VAP (43.8% vs. 31.3%, *p* = 0.189), despite the finding that patients with NVHAP had less severe illness at the onset of bacteremia and received appropriate antimicrobial therapy more often than patients with VAP. Multivariate logistic regression analysis was performed to identify potential independent risk or protective factors for 14-day mortality. Although ICU admission did not meet the criteria for entering multivariate logistic regression analysis, it was included because of its clinical importance. Patients with VAP caused by *A. baumannii* had a significantly lower mortality than those with NVHAP on regression analysis (odd ratio [OR] = 0.201; 95% confidence interval [CI] = 0.075-0.538; *p* = 0.001). Patients with higher APACHE II scores (OR = 1.164; 95% CI = 1.103-1.229; *p* <0.001), use of corticosteroids (OR = 5.739; 95% CI = 2.002-16.454; *p* = 0.001) and solid malignancies (OR = 4.242; 95% CI = 1.554-11.578; *p* = 0.005) also had a significantly higher 14-day mortality regardless of the use of ventilators. ICU admission was not found to be a protective factor for 14-day mortality.

## Discussion

In this study, we found that patients with *A. baumannii* bacteremia with NVHAP or VAP had similar demographic characteristics and Charlson comorbidity scores at hospital admission. Patients with NVHAP differed from VAP in that fewer acquired their infections in an ICU and they developed pneumonia sooner after admission. They received intensive care for pneumonia less often and had fewer invasive and surgical procedures. Patients with NVHAP also had lower APACHE II scores at the onset of bacteremia. They presented with bilateral lung involvement less often and had fewer antimicrobial resistant strains. The crude 14-day mortality rate was slightly higher in NVHAP patients than those with VAP. Following adjustment for multiple risk factors by logistic regression analysis, patients with VAP had lower 14-day mortality than those with NVHAP.

The findings on regression analysis appear to be counter-intuitive since patients with NVHAP were less ill and did not appear to require use of a ventilator. They would not be expected to be better off if treated with a ventilator, but this remains a possibility since it is difficult to assess individual clinical decisions in critically ill patients. It is reasonable to suppose that since most VAP patients were managed in ICUs, ventilator use may have been a surrogate marker for better and more intensive care. The upper airways of ventilated patients are better protected from contaminated secretions and their sputum is more readily expectorated [23,24]. The VAP patients might have been diagnosed and received antimicrobial therapy earlier.

Prompt use of appropriate antibiotics is essential to optimize the outcome of HAP [25-27]. A recent study showed that appropriate antimicrobial therapy reduced the mortality of bacteremic *A. baumannii* infections [11]. In the current study the isolates from patients with VAP were more likely than those with NVHAP to be resistant to multiple antimicrobial agents. This probably accounted for the tendency for less patients with VAP to receive appropriate therapy. Nevertheless, the frequency of antimicrobial resistance and inappropriate treatment was high in both groups. This finding is not surprising since *A. baumannii* is often resistant to the antimicrobial agents used for empirical therapy of patients with HAP. Routine respiratory surveillance cultures might be helpful to guide the selection of the most appropriate empirical therapy [28,29]. This should be particularly useful in institutions where multidrug resistant pathogens are endemic. The antimicrobial susceptibility of 1,160 strains of *Acinetobacter* species isolated during a 10-year nationwide survey in Taiwan was recently reported [30]. The overall susceptibility to tigecycline and colistin was 97.7% and 99.8%, respectively. The susceptibility of carbapenem resistant isolates to these drugs was 98.1% and 100% respectively [30]. More prompt use of these antimicrobial agents should improve use of appropriate antimicrobial therapy. These drugs were not available at our hospital during the study period.

It is also possible that there were important differences in the pathogenicity of the invading microorganism in the NVHAP and VAP populations. This concept is based on the higher frequency of antibiotic resistant strains among patients with VAP. It has been shown that the survival fitness and virulence of bacteria are often compromised in isolates with multidrug resistance [31,32]. PFGE analysis did not reveal a specific epidemic clone of *A. baumannii* among the two groups, but we cannot exclude the possibility that patients with VAP were infected by less invasive or less virulent clones. Another possible explanation is that patients with NVHAP may have been more susceptible to infection with *A. baumannii* than those with VAP because they developed bacteremic pneumonia without the need for intubation and mechanical respiration.

The APACHE II score at the onset of *A. baumannii* bacteremia has been shown to be a reliable parameter for predicting mortality [33]. In the current study we found that APACHE II score to be an independent risk factor for mortality in patients with *A. baumannii* HAP. In addition, solid malignancies and usage of corticosteroids were identified as independent risk factors for mortality in this study. The proportion of malignancies was higher in NVHAP patients (46% vs. 29%), though there was no significant difference between the two groups, but following regression analysis, solid malignancies was an independent risk factor for mortality. The higher proportion of malignancies may also play a role in the poorer outcome of NVHAP patients. The findings posed important information in risk stratification for *A. baumannii* HAP.

This is the first study specifically designed to compare the outcome of NVHAP and VAP bacteremic *A. baumannii* pneumonia. The strengths of this study are the relatively large number of patients managed in a major tertiary care teaching hospital using well defined criteria for underlying diseases, examination of clonal relationships and a clear 14-day end-point. The limitations include the difficulty to differentiate colonization from infection in sputum cultures of patients with HAP. To improve the specificity of the quantitative sputum cultures we excluded patients with *A. baumannii* bacteremia that could not be attributed to pneumonia. Concomitant catheter related bacteremia or secondary catheter infection could not be completely excluded because central venous catheter cultures were unavailable. Because of the retrospective design we were unable to control the selection of the antimicrobial agents prescribed by the hospital staff. However, there were no significant differences between the VAP and NVHAP in respect to the choice of drugs and variety of antimicrobial regimens that might have affected the clinical outcomes.

## Conclusions

Patients with bacteremic NVHAP and VAP caused by *A. baumannii* had similar crude mortality rates, but on logistic regression analysis those receiving ventilator assistance had a significantly lower mortality. This may have been due to better airway protection, more intensive monitoring with earlier diagnosis and treatment in patients with VAP, greater innate susceptibility to infection in those with NVHAP and differences in the virulence of *A. baumannii*.

## Abbreviations

A. baumannii: Acinetobacter baumannii; APACHE II: Acute Physiology And Chronic Health Evaluation II; ATS/IDSA: American Thoracic Society/Infectious Diseases Society of America; BAL: Broncho-alveolar lavage; CI: Confidence interval; CLSI: Clinical Laboratory Standards Institute; COPD: Chronic obstructive pulmonary disease; DNA: Deoxyribonucleic acid; HAP: Hospital-acquired pneumonia; ICU: Intensive care unit; NVHAP: Non-ventilated HAP; OmpA: Outer membrane protein A; OR: Odds ratio; PCR: Polymerase chain reaction; PFGE: Pulsed-field gel electrophoresis; SPSS: Statistical Package for the Social Sciences; TSGH: Tri-Service General Hospital; T-VGH: Taipei Veterans General Hospital; UPGMA: Unweighted pair-group method with arithmetic averages; VAP: Ventilator-associated pneumonia.

## Competing interests

Te-Li Chen is a medical advisor of TTY Biopharm. Other authors declare that they have no competing interests.

## Authors’ contributions

YSY, YTL and TLC participated in the study design, analysis of data, and writing of the manuscript. JRS and SCK participated in data collection. TWH and CHY participated in analysis of data. JCL, CPF and FYC revised the manuscript with important intellectual contribution. All authors read and approved the final manuscript.

## Pre-publication history

The pre-publication history for this paper can be accessed here:

http://www.biomedcentral.com/1471-2334/13/142/prepub
